# High physical activity is associated with decreased fungiform papillae area and number, elevated sucrose recognition thresholds, and increased IL-6 levels: an observational human study

**DOI:** 10.1186/s12986-025-01050-8

**Published:** 2025-11-28

**Authors:** Isabella Kimmeswenger, Marlies Gaider, Kevin Doppelmayer, Jakob P. Ley, Barbara Lieder

**Affiliations:** 1 Institute of Physiological Chemistry, Faculty of Chemistry, University of Vienna, Vienna, 1090 Austria; 2 Vienna Doctoral School in Chemistry (DoSChem), University of Vienna, Vienna, 1090 Austria; 3 Christian Doppler Laboratory for Taste Research, Faculty of Chemistry, University of Vienna, Vienna, 1090 Austria; 4 Symrise AG, 37603 Holzminden, Germany; 5 Institute of Clinical Nutrition, University of Hohenheim, 70599 Stuttgart, Germany

**Keywords:** Taste perception, Sucrose recognition threshold, Dietary behavior, Low-grade inflammation, Physical activity, IL-6, Fungiform papillae

## Abstract

**Background:**

Disease-related inflammation affects chemosensory signaling, but knowledge on the impact of exercise-induced low-grade inflammation on taste function remains scarce. Here we hypothesized that intense habitual physical activity modifies sweet taste perception via increased cytokine release.

**Methods:**

In an observational human study we compared participants (m/f) engaging in high (*n* = 34) and low (*n* = 31) levels of habitual physical activity. Salivary IL-6 and urinary 8-iso-prostaglandin F2α levels, body composition, sucrose recognition threshold, preference and consumption of sweet foods, size and area of fungiform papillae as well as selected hormones regulating food intake were recorded. Statistical analysis was conducted using Principal Component Analysis (PCA) followed by Student’s t-tests and multiple regression models.

**Results:**

The PCA summarized the main outcome variables to two principal components (PC). PC1 was primarily influenced by body composition and fungiform papillae markers, while sucrose recognition thresholds, sweet food consumption, and IL-6 levels strongly contributed to PC2. Compared to the low activity group, the high activity group showed on average an increased sucrose recognition threshold (+ 35.8 ± 12.8%), increased IL-6 concentrations (+ 25.6 ± 10.9%), higher consumptions of sweet foods (+ 18.8 ± 4.9%) and decreased number (­24.8 ± 4.9%) and area (-29.8 ± 6.4%) of fungiform papillae.

**Conclusions:**

The association between modified sweet taste function markers and increased IL-6 levels suggests that inflammatory processes may contribute to exercise-related changes in chemosensory perception.

**Trial registration:**

The study was approved by the Ethics Committee of the University of Vienna (approval number 01125) and registered at ClinicalTrial.gov (NCT06384365).

**Graphical abstract:**

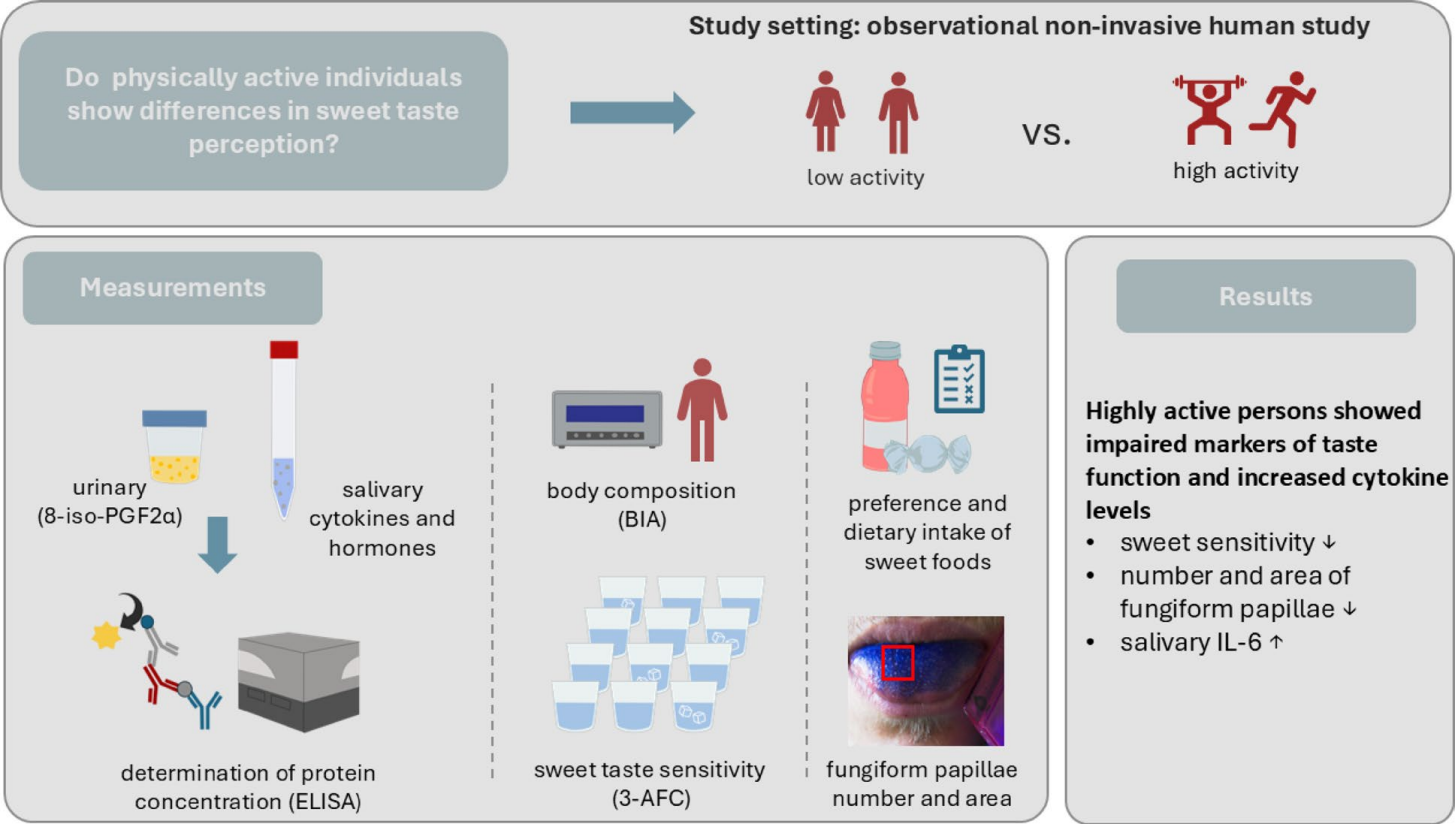

**Supplementary Information:**

The online version contains supplementary material available at 10.1186/s12986-025-01050-8.

## Introduction

Taste perception varies intra- as well as interindividually [1], and since taste is a key driver of food choices [2], understanding the plasticity of taste perception is of high importance for understanding nutritional behavior. This is particularly relevant for sweet taste perception, as high sugar intake poses cardiometabolic risks [3] and should be limited to 10% of daiy caloric intake [4, 5]. Next to sex, age and pathological conditions, taste perception is also influenced by modifiable lifestyle factors [1, 6], one of them being physical activity.

Studies examining effects of acute physical activity on sweet taste have reported mixed findings, with effects ranging from no change to increased sweet taste sensitivity and enhanced sweet taste preferences following exercise [7–10]. Although the influence of habitual physical activity on taste perception has been less studied, research by Feeney et al. [11] provides insight into this relationship. Within WHO-recommended activity levels of 150–300 min per week [12], they showed that moderately active individuals (>2.6 h/week) perceived a high sucrose concentration as significantly more intense than less active individuals. Such an enhancement in the intensity of sweet taste perception could promote earlier sensory saturation, potentially supporting a limited sugar intake. This dietary benefit would align with other positive effects of moderate physical activity [13–17], including reductions in inflammatory markers such as IL-6, C-reactive protein (CRP), and TNF-α [18–22]. However, when physical activity substantially exceeds recommended levels, its effects on inflammatory processes show a different pattern. Intense, prolonged exercise can increase inflammatory mediators compared to moderate exercise [23], as also confirmed in elite athlete studies [24, 25]. This response can result from muscle damage, trauma or inadequate recovery [26–28]. Inflammation is known to impair taste perception in various pathological contexts including autoimmune conditions such as lupus [29] and rheumatoid arthritis [30], infectious diseases as for example HIV [31] and COVID-19 [32], as well as metabolic disorders such as obesity [33]. In a previous study, we found that male subjects with higher sucrose recognition threshold and higher sweet preference had lower body fat content, suggesting higher levels of physical activity [34]. Accordingly, we recently proposed that low-grade inflammation induced by exercise may similarly affect taste perception [35]. We speculate this represents an adaptive mechanism where reduced sweet taste sensitivity facilitates consumption of larger amounts of energy-dense sweet foods needed to meet the elevated metabolic demands of intense physical activity without sensory saturation.

Therefore, we addressed the hypothesis that extensive habitual physical activity leads to inflammation, a decrease in sweet taste perception and consequently increased sweet preference and consumption of sweet foods [35].

To test our hypothesis, we conducted an observational study comparing sweet taste perception and associated markers between low (≤ 150 min/week) and high (≥ 360 min/week) physical activity groups, based on WHO guidelines [12]. We measured sucrose recognition thresholds (as a measure for sweet taste sensitivity), preferences for sweet foods, sweet food consumption, inflammatory markers (salivary IL-6, urinary 8-iso-prostaglandin F2α (8-iso-PGF2α)), body composition, and fungiform papillae (FP) morphology and determined group differences and associations between biomarkers. IL-6 was selected as the primary inflammatory parameter due to its prominent role in exercise-induced inflammation [36], and its release in correlation to carbohydrate availability, additionally suggesting energy sensor functions [37, 38], while 8-iso-PGF2α served as a marker of oxidative stress.

## Methods

### Study design

This observational non-invasive human study was approved by the Ethics Committee of the University of Vienna (approval number 01125), registered at ClinicalTrial.gov (NCT06384365) and conducted in accordance with the principles of the Declaration of Helsinki. All volunteers signed a written informed consent and data privacy guidelines prior to participation and were remunerated for the time they devoted to the study. Based on results of a preliminary analysis, where we found a correlation between body fat (as a marker for physical activity) and sweet preference, a power analysis using the software GPower 3.1.9.7 [39] determined 62 participants (m/f) with an effect size of 0.33 (power of 0.85, alpha = 0.05). To analyze the effect of physical activity on parameters related to sweet taste perception participants for a low and a high activity group were recruited (31 per group). Criteria for activity groups followed WHO guidelines [12]. Accordingly, in the low activity group, participants were selected based on performing ≤ 2.5 h of habitual physical activity per week and ≥ 6 h per week distributed across a minimum of three days for the high activity group. Participants were required to have been engaged in their respective activity levels for at least 6 months prior to participation. Test persons further had to be 18–45 years, body mass index 18.5–29.99 kg/m² and body fat < 30% (male) or < 35% (females). Exclusion criteria were alcohol/drug addiction, tobacco consumption, anti-inflammatory medication and antibiotics in the last 3 months, metabolic diseases requiring drug therapy, anosmia/ageusia, infections in the last 3 weeks, pregnancy and breastfeeding, known allergies or refusal to consume the foods used, inflammatory diseases of the gums or oral cavity.

Interested candidates received an online questionnaire covering inclusion criteria and questions regarding extent and distribution of physical activity. Participants meeting inclusion criteria were invited for a study day.

Participants arrived at the research facility in the morning after a 10-hour fasting period. Additionally, participants were instructed to refrain from any physical activity 24 h prior to the investigation to minimize acute exercise-induced effects on sensory measurements and biomarker concentrations. They were asked to provide a urine sample for urinary glucose screening (*Medi-Test Combi 3 A*,* #133553*,* Macherey-Nagel*). Additionally, urine samples of female participants were used for pregnancy screening (*Clinitest hCG pregnancy test*,* #133561*,* Siemens Healthcare*). To record BMI [kg/m2], height [m] was measured with a stadiometer (*Seca; max: 2.10 m; accuracy 0.01 m*) and body weight [kg] with a digital scale (*SOEHNLE person scale Silver Sense (61350)*,* max: 150 kg; accuracy; 0.1 kg*). To exclude manifest hypertension, blood pressure was measured using an upper arm blood pressure monitor (*Medisana*,* BU 512*).

### Anthropometric measurement

Body composition was measured using a bioelectrical impedance analysis (BIA) device (*Impedanz Analysator*,* Nutriguard MS*,* 16340238*,* Data Input GmbH*), with suitable electrodes (*Elektroden*,* 821*,* BIANOSTIC AT*), according to the manufacturer’s instruction manual. The software NutriPlus (version 7.0, Data Input GmbH) was used for measurement analysis. The parameters body cell mass (BCM) and body fat were used to analyze body composition.

### Measurement of salivary and urinary biomarkers

Participants provided 2 mL of unstimulated saliva using a paper straw to facilitate the transfer into a reaction tube. The reaction tubes were immediately placed on ice. Samples were split into two 1 mL aliquots. One aliquot was frozen immediately for IL-6 and serotonin analysis. The remaining 1 mL was centrifuged (800 rpm,10 min, 4 °C). The supernatant was decanted and aspirated with a 2 mL syringe cannula. Samples were aliquoted further and stored at −80 °C for analysis of TNF-α, glucagon, glucagon-like peptide-1 (GLP-1) and leptin. The selection of saliva types for specific parameter analyses was based on preliminary testing for IL-6 and TNF-α and guided by literature for remaining parameters [40–42]. For analysis samples were thawed on ice and analyzed using standardized ELISAs with horseradish peroxidase (HRP) reporter enzyme and tetramethylbenzidine (TMB) detection according to manufacturer’s instructions. Most assays used sandwich ELISA format, except serotonin which employed competitive ELISA format. Standard curves were generated for each assay using provided standards. The following kits were used: Human IL-6 ELISA Kit (*Abcam*), Human TNF alpha ELISA Kit (*Abcam*), Human Glucagon ELISA Kit (*Thermo Fisher Scientific*), GLP-1 Total ELISA Kit (*Merck KGaA*), Serotonin High Sensitive ELISA (*DLD Diagnostika*) and Human Leptin ELISA Kit (*Abcam*).

Provided urine samples were immediately placed on ice. Urine was aliquoted and suspended with 10 µg/mL indomethacin (*Indomethacin*,* Merck*) as a prostaglandin synthetase inhibitor. Samples were stored at −20 °C for a few hours and then at −80 °C until analysis. The determination of 8-iso-PGF2α was performed using a standardized competitive ELISA with alkaline phosphatase (AP) reporter enzyme and pNpp substrate detection (*8-iso-PGF2 alpha ELISA Kit*,* Abcam*) according to manufacturer’s protocol. Standard curves were generated using provided standards. Urine samples were thawed on ice and the assay was performed with readings at 405 nm. Furthermore, creatinine concentrations of each sample were determined for normalization of 8-iso-PGF2α, using a colorimetric assay performed according to the manufacturer’s instructions (*Creatinine urinary Colorimetric Assay Kit*,* Cayman*).

### Assessment of sweet taste perception

For assessing the participants’ sweet taste sensitivity, the individual sucrose recognition threshold was determined using a 3-alternative forced-choice test (3-AFC). The test model was based on DIN EN ISO 3972:2013-12 [43] but included two additional concentrations to ensure more precise results. The test involved tasting sample triplets, whereby two of these were tap water and one sample was a sugar solution, consisting of sucrose (*Wiener Zucker*,* 342.30 g/mol*) dissolved in tap water. The concentration of the sugar solution was increased for each sample triplet [0.34, 0.55, 0.94, 1.56, 2.59, 4.32, 5.76, 7.2, 9.6, 12 g/L] and participants were asked to indicate the sample differing from water for each triplet and further specify the differing taste quality and their sureness concerning their choice. The concentration at which participants first recognized the sweet solution within the triplet intentionally represents the sucrose recognition threshold. Test solutions were provided at room temperature in 20 mL plastic beakers containing a sample size of 10 mL and labeled with a randomly assigned three-digit code. Participants were asked to neutralize (rinse) with tap water in between triplets.

### Assessment of sweet preference and consumption of sweet foods

Participant’s sweet preference was assessed by means of three different methods: a validated questionnaire, a tasting of foods with different concentrations of sugar and a single-blinded naturalistic choice experiment with full disclosure provided after study completion. Results of the different methods were summarized by carrying out a factor analysis to obtain one sweet preference factor which was used for further statistical analysis.

#### Questionnaire

The questionnaire focused on the liking of sweet and sweet-fatty foods. It was based on the extensively validated PrefQuest questionnaire by Deglaire et al. [44], which demonstrated excellent psychometric properties in a large-scale study (*n* = 47,803) with robust construct validity, internal consistency, and convergent/divergent validity for assessing sweet preference. The questionnaire has been minimally adjusted to Austrian dietary habits with culturally appropriate food substitutions and consisted of 4 parts.

1. How much one likes certain foods (9-point category scale ranging from “I do not like it at all” to “I like it very much”),
2. How much of a certain sweet-tasting food one likes best when eating selected meals (participants can choose from different pictures showing dishes with increasing amounts of sweet foods),
3. Choice of drinks when eating out (choice of different sweet and non-sweet drinks)
4. Questions about sweet-related eating behavior to assess social and psychological aspects (choice of different answer options in increasing order).

Participants rated taste preferences independently of actual consumption patterns. Parts 1, 2, and 4 used numerical scales (lowest sweet preference = 1). Part 3 scored sweet drinks as 1, non-sweet as 0 (3 choices each). An overall liking score combined all categories.

#### Tasting

Participants were offered four samples with varying amounts of added table sugar for two chosen food items. The foods were puréed strawberries, as an example for an “only sweet” food and chocolate pudding enriched with whipped cream (70% *lactose-free whole milk* + 30% *lactose-free whipped cream*) as an example for a sweet-fatty food. Food items were obtained at a local supermarket. Samples were prepared the day before the experiment and portioned for the participants one hour before the tasting. Composition of the samples can be obtained in Table 1.

**Table 1:** Composition of food items to assess sweet preference. C_final_ = final concentration

	Strawberry puree			Chocolate pudding		
	Strawberries [g]	Added sucrose [g]	C_final_ sucrose [%]	Chocolate pudding [g]	Added sucrose [g]	C_final_ sucrose [%]
Sample 1	100	0	4.6	100	0	3
Sample 2	95	5.6	10	90	7.3	10
Sample 3	90	10.9	15	80	17.6	20
Sample 4	85	16.1	20	70	27.9	30

Participants first received four samples of the strawberry puree and afterwards four samples of the chocolate pudding. Samples were provided in 20 mL plastic beakers and in randomized order. Participants were asked to neutralize their mouth with tap water between the different food items and were allowed to neutralize between the different samples. They could taste the different samples as often as they wanted and were asked to choose the sample which they liked the most.

#### Observation of naturalistic choice

The study incorporated a naturalistic choice experiment, with full disclosure provided after study completion. They were offered a selection of sweet and savory baked goods (donuts, pretzels, muffins, cheese breadsticks, gratinated bread, and danish pastries), which they could either consume immediately or take away. Sweet baked goods were defined as containing at least 10% natural sugar, while savory items contained less than 5% natural sugar. The participants’ choices were documented and analyzed to assess sweet food preferences. This approach was chosen to capture natural behavior and reduce social desirability response bias [45].

Dietary habits concerning sweet foods were assessed by the means of a Food Frequency Questionnaire (FFQ) as described previously [35].

### Assessment of physical activity

For a detailed analysis of participants’ physical activity behavior an International Physical Activity Questionnaire (IPAQ) in the long form was performed. This self-reported measurement has shown reliability and validity within different contexts [46, 47]. The derived data can be converted into metabolic equivalents of task (METs) to quantify total physical activity per week, which was performed according to the IPAQ scoring system.

### Analysis of FP

Participants were asked to rinse their mouths with water and swallow the remaining saliva. Then a commercially available plaque staining solution containing the dyes CI 45410 and CI 42090 [*Mira-2-Ton (Miradent*,* PZN 3057408)*,* Hager & Werken*] was applied to the tip of the participants tongue using a sterile cotton swab. Pictures of the tongue were analyzed using a two-step image analysis protocol with GIMP 2.10.36 and ImageJ 2.14.0. First, in GIMP, the ruler visible in each photograph was used to establish scale calibration. A 1 cm² area of interest was precisely delineated 0.5 cm away from the tip of the tongue on the participants’ right tongue side using the ruler for measurement reference. Within this defined area, individual FP were manually identified and circled based on their characteristic morphological features after staining: larger size, rounded shape, and lighter coloration compared to the smaller, pointed, blue-stained filiform papillae [48]. All identified FP circles were then filled with a uniform color to create binary markers. The processed image was exported and imported into ImageJ 2.14.0 for quantitative analysis. ImageJ was used to calculate: (1) the total number of colored regions (FP number), and (2) the percentage of the 1 cm² area covered by colored regions (FP area). This approach provided both absolute counts and relative area measurements of FP within the standardized area of interest.

**Fig. 1:**
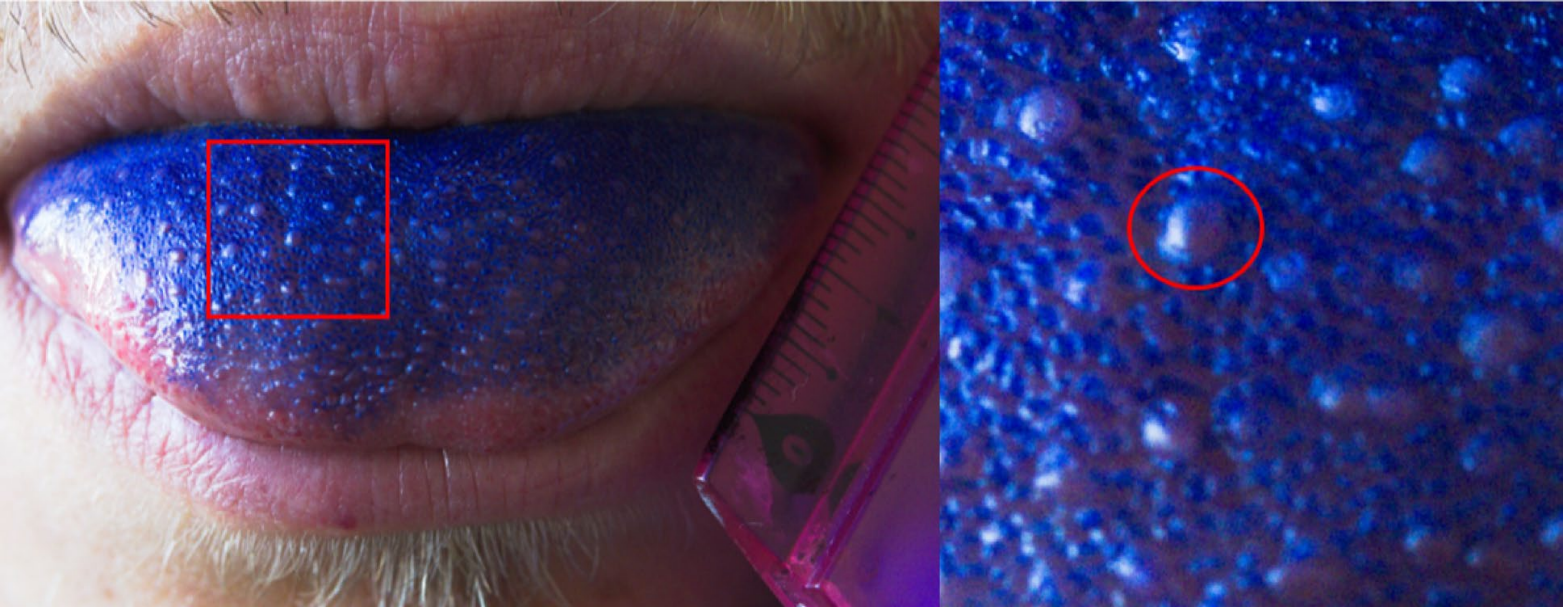
Representative picture of the anterior part of a human tongue, stained with CI 45410 and CI 42090 solution. The filiform papillae exhibit a blue color, allowing to distinguish from the FP

A representative picture can be seen in Fig. 1.

### Statistical analysis

Statistical analyses were conducted using GraphPad Prism (version 10.2.3), SigmaPlot (version 14.5) and IBM SPSS Statistics (version 29.0.0.0). Data was tested for normal distribution using Shapiro-Wilk test. To reduce dimensionality of sweet preference variables, a main component analysis using an extraction method based on eigenvalues was performed. A Principal Component Analysis (PCA) with standardized method was carried out with the variables IL-6, 8-iso-PGF2α, FP number and area, sucrose recognition threshold, BCM, body fat, sweet consumption, and sweet preference. Principal components (PCs) were retained according to parallel analysis criteria. Group comparisons were assessed by Students t-test with two-tailed p-value and Mann-Whitney-U test for non-normally distributed data, respectively.

Multiple linear and ordinal regressions were performed to analyze connections between PC variables as well as the involvement of leptin, GLP-1, glucagon, serotonin and TNF-α in hypothesized mechanisms. Models were corrected for sex, age and BMI.

## Results & discussion

**Fig. 2:**
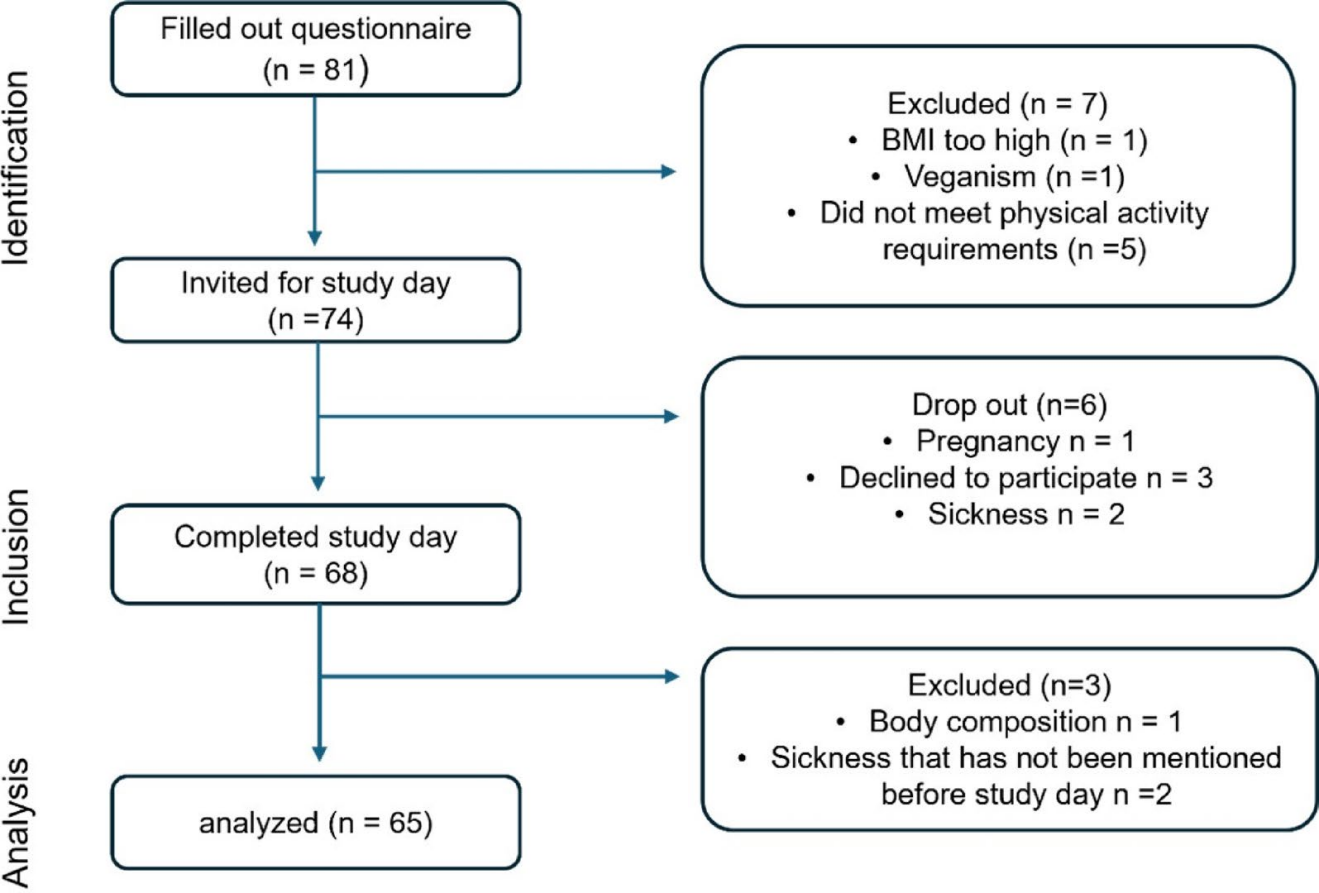
STROBE Flow Chart Diagram

A total of 74 participants met the inclusion criteria and were invited to the study day. Of these, 68 participants completed the study, and 65 were included in the final analysis (Fig. 2).

The 65 participants were almost equally distributed between low activity (*n* = 31) and high activity (*n* = 34) groups with a comparable distribution of sexes. Participant’s main characteristics are summarized in Table 2 and further outcome parameters analyzed in the course of the study can be found in table S1.

**Table 2:** Demographic and anthropometric characteristics of the study participants

	Total cohort	≤ 2.5 h physical activity	≥ 6 h physical activity	Group difference [*p*-value]
**Number of participants**	65	31	34	
**Female**	33	16	17	
**Male**	32	15	17	
**Age [years]**	27 (19–44)	28 (22–44)	26 (19–33)	< 0.05
**Height [cm]**	173.2 (158.7–193.0)	173.1 (158.7–193.0)	173.3 (159.2–193.0)	n.s.
**Weight [kg]**	66.9 (50.4–90.2)	64.2 (50.4–89)	69.3 (54.7–90.2)	n.s.
**BMI [kg/m²]**	22.2 (18.5–27.8)	21.3 (18.6–25.5)	23.0 (18.5–27.8)	n.s.

Mean values (min – max). BMI = body mass index

### Differences in physical activity between groups can be accounted to IPAQ leisure-time domain

To validate group assignment we assessed physical activity behavior using the validated self-report questionnaire IPAQ [46, 47].

The high activity group had higher total physical activity (+ 4840 MET-min/week, *p* < 0.001). This difference occurred only in the leisure-time domain (*p* < 0.0001), reflecting participants’ exercise-related activities. Both groups demonstrated comparable activity in work, transportation, and domestic domains, suggesting similar baseline activity levels in daily life. The difference in total physical activity was therefore primarily explained by exercise engagement rather than occupational physical demands. These findings confirm the appropriate classification of participants into activity groups to assess the associations between exercise and taste perception (Table 3).

**Table 3:** Participants’ physical activity scores for total physical activity and subcategories assessed by IPAQ questionnaire

	< 2.5 h physical activity/week	> 6 h physical activity/week	Group difference [*p*-value]
**Total Physical Activity Score [MET-min/week]**	mean = 3991 median = 3332 min-max = 554–11591	mean = 9890 median = 8172 min-max = 707–32527	< 0.0001
**Work domain** **[MET-min/week]**	mean = 1292 median = 233 min-max = 0–8352	mean = 3069 median = 636 min-max = 0–22945	n.s.
**Active transportation domain [MET-min/week]**	mean = 1079 median = 855 min-max = 66–3927	mean = 1510 median = 1386 min-max = 0–5814	n.s.
**Domestic and garden domain [MET-min/week]**	mean = 606.3 median = 360 min-max = 0–3060	mean = 771 median = 395 min-max = 0–4500	n.s.
**Leisure-time domain [MET-min/week]**	mean = 1046 median = 960 min-max = 99–3759	mean = 4439 median = 3966 min-max = 37–18106	< 0.0001

Differences between groups were analyzed by Mann-Whitney-U test. MET = metabolic equivalent of task

### Relationship of variables related to physical activity and sweet taste perception

**Fig. 3:**
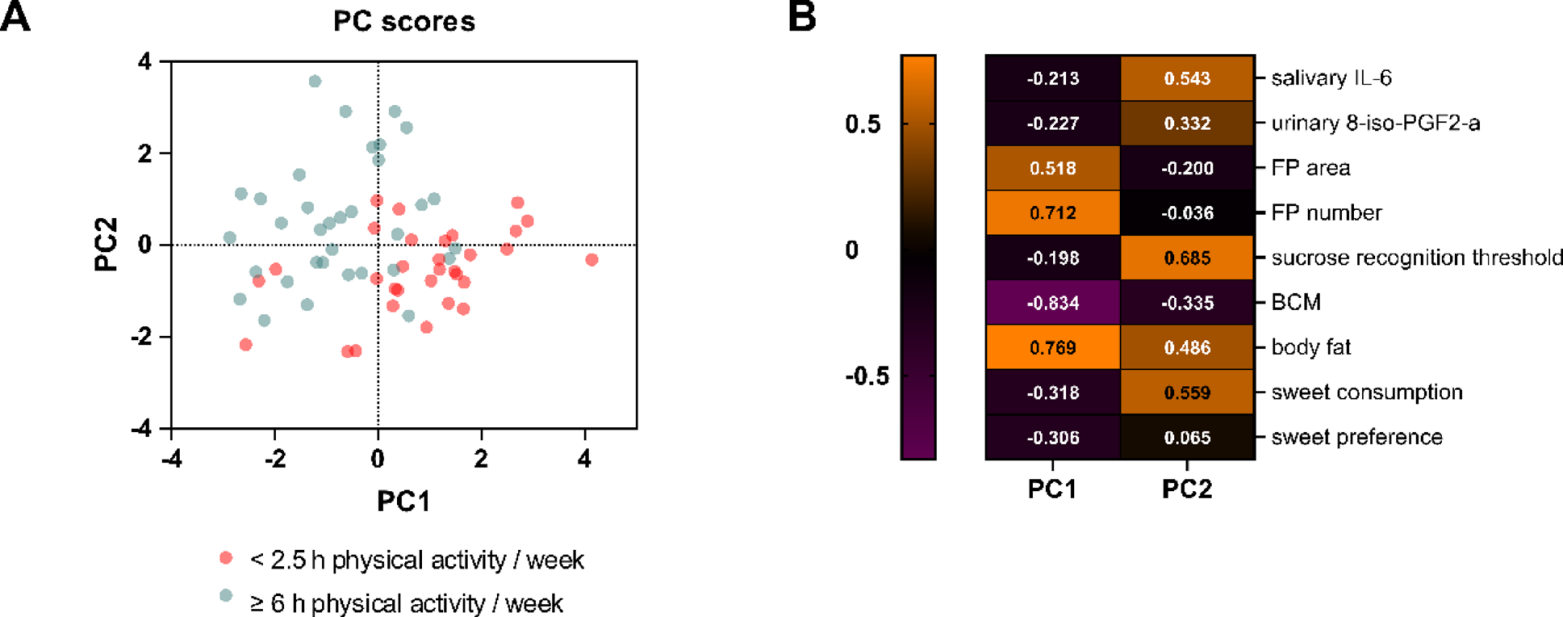
Results of a Principal Component Analysis (PCA) conducted by standardized method. Principle Components (PC) have been selected based on parallel analysis. (**A**) Scoreplot of individual participants’ scores on Principal Component 1 (x-axis) and Principal Component 2 (y-axis), with colors indicating low (blue) and high (red) physical activity groups. Origin represents average component values. (**B**) Heatmap showing loading scores of the variables for the selected PCs. PC1 = Principal Component 1, PC2 = Principal Component 2, IL-6 = Interleukin 6, 8-iso-PGF2α ­ 8-iso-prostaglandin F2α, FP = fungiform papillae, BCM = body cell mass

Principal Component Analysis (PCA) explored multivariate relationships in the dataset. PCA explained 44.15% of cumulative proportion of variance by two principal components (referred to in the graph as PC1 and PC2), a value that can be considered as reasonable in nutritional and behavioral research [49–51]. The PCA plot (Fig. 3A) demonstrates separation between the low (< 2.5 h/week) and high activity (≥ 6 h/week) groups along Principal Component 1 (PC1), indicating a strong association with physical activity. For Principal Component 2 (PC2), activity groups were differently distributed. The high activity group was more vertically dispersed, indicating that PC2 captures variation beyond physical activity.

PC1 explained 26.58% variance and was primarily influenced by body composition and FP. Loading scores corresponded to −0.834 for body cell mass (BCM), 0.769 for fat mass as well as 0.712 for number and 0.518 for area of FP. PC2 explained a further 17.57% variance and sucrose recognition threshold (0.685), consumption of sweet foods (0.559) and salivary concentration of IL-6 (0.543) had the strongest loading scores.

An overview of the loadings (coefficients indicating how strongly each original variable contributes to each principal component) contributing to the two PCs can be found in Fig. 3B. 8-iso-PGF2α (PC1: −0.227, PC2: 0.332) and sweet preference (PC1: −0.306, PC2: 0.065) did not show strong loading scores. While there was no difference in sweet preference between activity groups, urinary 8-iso-PGF2α was elevated by 88.3% in the high activity group (*p* < 0.05) and correlated with physical activity (*r* = 0.51, *p* < 0.0001), confirming group classification.

Having identified these distinct patterns through our exploratory analysis, we subsequently examined individual variables, showing the strongest loading scores within each principal component, through group comparisons and hypothesis-driven regression analysis.

### Decrease in FP area and number is associated with high physical activity level

**Fig. 4:**
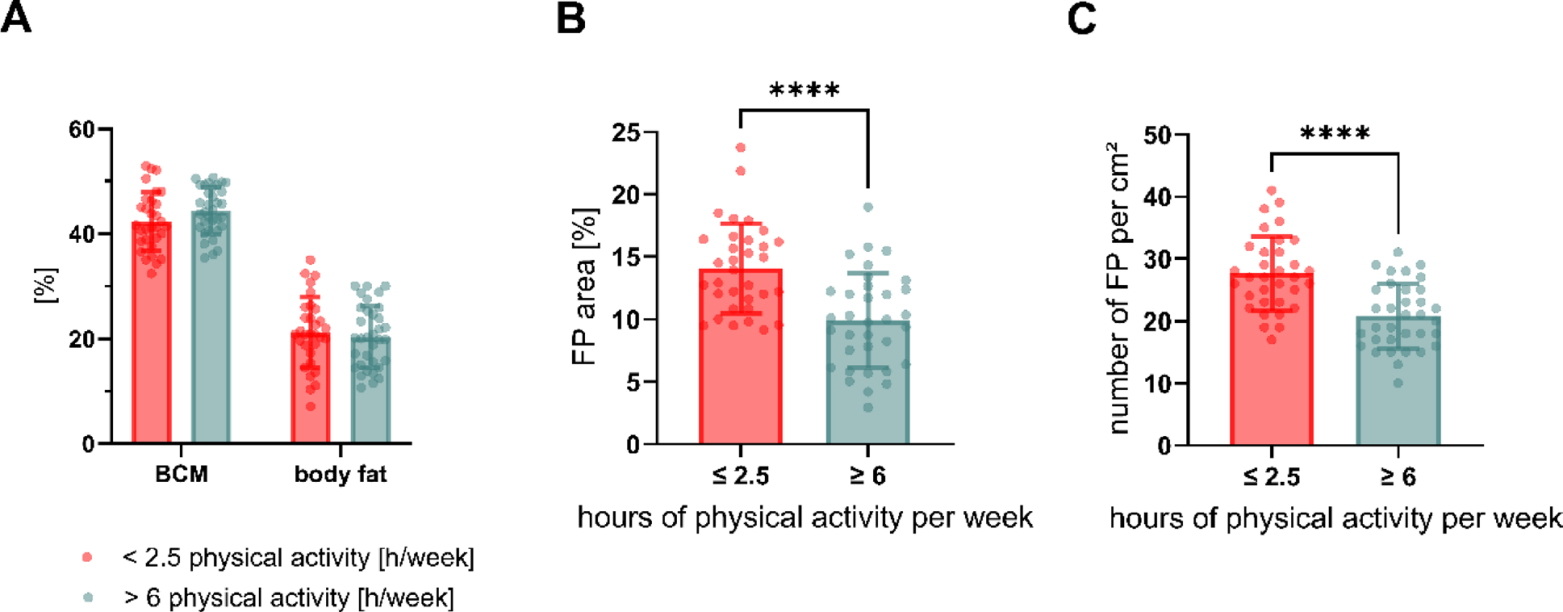
Analysis of variables strongly contributing to PC1 and their association with physical activity. **(A)** BCM (body cell mass) and body fat in % between activity groups. **(B)** Differences in FP (fungiform papillae) area in % and **(C)** number of FP. Group Comparisons were analyzed using unpaired t-test and two-tailed p-value, data is displayed as mean ± SD and individual data points. *n* = 65 (n per group = 31–34), **** indicates significant difference with *p* < 0.0001

PC1 was mostly influenced by body composition and FP. We did not detect differences in BCM or fat mass between the activity groups (Fig. 4A). We attribute this finding to the large variance in our data set, resulting from the mixed-sex composition of our study population. However, FP area 29.8 ± 6.4%, *p* < 0.0001, Fig. 4B) and FP number (−24.8 ± 4.9%, *p* < 0.0001, Fig. 4C) were significantly lower in the high activity group. To our knowledge, this represents the first evidence that habitual intense physical activity is associated with reduced fungiform papillae number and area in humans. Multiple linear regression revealed that the FP number, but not area, was significantly influenced by body fat (β = 0.621, 95% CI [0.002, 1.240], *p* < 0.05, Supplementary Table S2-3). A higher FP number was associated with higher body fat after controlling for sex and age. This appears contradictory to results reported by Kaufman et al., who demonstrated a negative association between FP area and neck circumference (an obesity marker) [52]. However, this discrepancy may reflect differences in study populations, as Kaufman et al. investigated a sample that included obese participants while our study focused on normal weight individuals with lower body fat percentages. Additionally, the direction of causality in the relationship between body composition and FP characteristics remains unclear. While our cross-sectional design limits causal inference, the positive association between body fat and FP number detected in our study may reflect different mechanisms than those operating in obesity.

To investigate a potential mechanism between body composition and FP, we subsequentially analyzed fasted salivary leptin, an adipokine released proportional to body fat [53, 54]. It has furthermore been demonstrated that leptin is associated with taste signaling, although studies have reported various effect directions [55]. In our study population participants in the high activity group had 31.8 ± 13.5% lower salvary leptin concentrations (*p* < 0.05). When leptin was added to the regression model for FP number, body fat (β = 0.619, 95% CI [0.298 0.941], *p* < 0.0001) and leptin (β = 0.209, 95% CI [0.000, 0.419], *p* < 0.05, Supplementary Table S3) were both significant, without correlating with each other (*p* >0.05), suggesting that they contribute independently to FP number. Previous studies have reported contradictory findings regarding the relationship between salivary leptin and body composition. While some studies found a strong correlation between leptin concentrations in saliva and plasma [42, 56], and between salivary leptin and measures of body composition [57], several research groups found no associations [42, 58, 59]. This suggests that a potential reason for the lack of correlation between leptin and body fat in our study population could be the sample material.

To summarize, our findings show that highly active participants had a decreased FP number which was influenced by a reduction in body fat and salivary leptin levels, possibly contributing independently to changes in FP morphology.

### High physical activity is associated with elevations in sucrose recognition threshold, IL-6 and consumption of sweet food

**Fig. 5:**
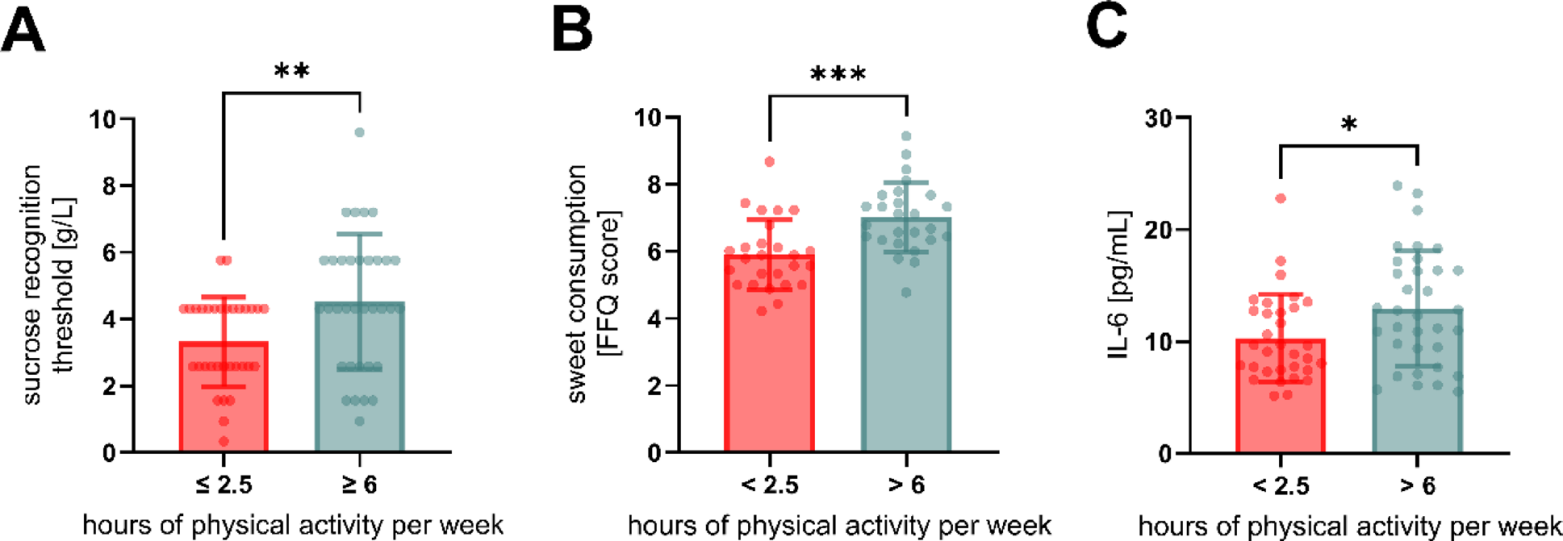
Analysis of variables strongly contributing to PC2 and their association with physical activity. **(A)** Differences in sucrose recognition threshold [g/L], **(B)** consumption of sweet foods [FFQ score] and **(C)** concentrations of fasted IL-6 in saliva [pg/mL] between activity groups. Group Comparisons were analyzed using unpaired t-test and two-tailed p-value, data is displayed as mean ± SD and individual data points. *n* = 65 (n per group = 31–34), * indicates significant difference with *p* < 0.05, ** *p* < 0.01 and *** *p* < 0.001

The sucrose recognition threshold, the consumption of sweet foods, and the salivary concentration of IL-6 showed the strongest loading scores for PC2. Comparisons between activity groups revealed that the high activity group had a higher sucrose (sweet taste) recognition threshold, indicating a reduced sweet taste sensitivity, compared to the low activity group (+ 35.8 ± 12.8%, *p* < 0.01. Figure 5A). This aligns with a previous study showing higher recognition thresholds in participants with lower body fat and higher resting metabolic rate [34], both outcomes of regular physical activity [60, 61]. Contrary, Feeney et al. showed that more active individuals perceived a high sucrose concentration as significantly sweeter than less active individuals [11]. However, the more active group (>2.6 h physical activity/week) in the study by Feeney et al. significantly differed in the activity level from our high activity group (>6 h/week). This suggests that the relationship between taste sensitivity and exercise level may not be linear, with moderate levels potentially enhancing sensitivity while very high levels impairing it. A curvilinear relationship was also found for the correlation between habitual physical activity and the consumption of sugar, with highly active participants consuming significantly more sugar compared to moderately active participants [62]. Our results support this relationship, as highly active participants reported a significantly higher consumption of sweet food (+ 18.8 ± 4.9%, *p* < 0.001, Figur 5B). According to our hypothesis, these differences in sucrose recognition threshold and sugar consumption might be attributed to increased IL-6 levels, as high physical activity levels can promote the secretion of IL-6 [23]. Correspondingly, in the present study, the high activity group had elevated concentrations of fasted salivary IL-6 (+ 25.6 ± 10.9%, *p* < 0.05, Fig. 5C) Multiple ordinal regression revealed the sucrose recognition threshold was significantly influenced by IL-6 (β = 0.117, 95% CI [0.002, 0.236], p = 0.045, Supplementary Table S4) after controlling for age, sex and BMI. Furthermore, we detected a trend for the sucrose recognition threshold contributing significantly to the consumption of sweet foods (β = 0.178, 95% CI [−0.021, 0.378], p = 0079, Supplementary Table S5). These results indicate an association between higher oral IL-6 levels and increased sucrose taste recognition thresholds, which in turn suggest an association with higher consumption of sweet foods as measured by FFQ. Future studies should validate these findings in larger populations and distinguish whether higher sweet consumption reflects specific sweet food seeking or higher overall energy intake in active populations.

While acknowledging the need for further validation, we hypothesize that the detected relationship between the variables suggests that an exercise-induced modification of sucrose recognition thresholds may be mediated by IL-6 and facilitates the consumption of larger amounts of energy-dense sweet foods needed to meet the elevated metabolic demands of intense physical activity without sensory saturation.

### IL-6 associations with sweet taste sensitivity involve inflammatory rather than metabolic processes

Our results show that IL-6 significantly contributes to sweet taste perception in our study population. However, as IL-6 is known to exert both pro- and anti-inflammatory effects [63] and may also function as an energy sensor [36], we systematically tested whether the influence of IL-6 on the sucrose recognition threshold is related to inflammatory or metabolic processes. Hence the satiety and glucose metabolism-regulating hormones glucagon, GLP-1 and serotonin were analyzed as energy sensor markers and TNF-α as a marker of inflammatory effects in a subsequent analysis. The metabolic markers were additionally shown to have established relationships with sweet taste perception [41, 64, 65].

Competitive regression modeling (in which biomarkers are tested pairwise with IL-6 to determine whether they compete for the same variance) revealed distinct patterns. When TNF-α was included alongside IL-6, the IL-6 effect weakened (β = 0.082, *p* = 0.198 vs. baseline β = 0.117, *p* = 0.045), while TNF-α showed a trend towards significance (β = 0.029, *p* = 0.076). This pattern indicates shared variance between IL-6 and TNF-α, consistent with common inflammatory pathway activation [29]. In contrast, metabolic markers (GLP-1, glucagon, serotonin) did not reduce IL-6’s power, with IL-6 maintaining or strengthening its effect when these variables were included, indicating that IL-6 and the metabolic marker have an independent relationship to the sucrose recognition threshold. The competitive analysis, supported by the correlation and the low p-value between IL-6 and TNF-α (*r* = 0.39, *p* = 0.001), suggests that IL-6 affects the sucrose recognition threshold through inflammatory rather than metabolic signaling (Table 4). The distinction between inflammatory and metabolic pathways is based on biomarker associations rather than anatomical specificity, as salivary measurements can reflect both local and systemic sources.

**Table 4:** Competitive regression analysis examining IL-6’s influence on the sucrose recognition threshold

Model	IL-6 [β]	IL-6 [t-value]	IL-6 [*p*-value]	Co-variable	Co-variable [*p*-value]	Interpretation
**Baseline**	0.117	1.929	0.045	-	-	Reference
**+ TNF-α**	0.082	1.289	0.198	TNF-α	0.076	Shared pathway
**+ GLP-1**	0.116	1.727	0.084	GLP-1	0.839	Independent
**+ Glucagon**	0.124	2.015	0.044	Glucagon	0.633	Independent
**+ Serotonin**	0.121	1.986	0.047	Serotonin	0.778	Independent

Multiple regression models tested IL-6’s contribution to the sucrose recognition threshold with different co-variables to explore the role of IL-6 in sweet taste sensitivity. Multiple Ordinal Regression Analysis controlled for age sex, and BMI. n = 64–65. β = unstandardized beta coefficient, IL-6 = Interleukin 6. TNF-α = tumor necrosis factor-α, GLP-1 = glucagon-like peptide-1

### FP characteristics were not associated with inflammatory markers and the sucrose recognition threshold

The number and area of FP were not related to sucrose recognition thresholds in our study population and also previous research on FP-taste relationships showed mixed results [66–70]. In our study, FP number weakly correlated with sucrose recognition thresholds in the low activity group (*r* = 0.38, *p* < 0.05), without being related to salivary IL-6 concentrations, but showed no association in the high activity group (*r* = 0.07, *p* >0.05). This suggests that the relationship between the FP area and the sucrose recognition threshold may exist in the general population but may be disrupted by high physical activity levels.

While Kaufman et al. showed that obesity-induced low-grade inflammation reduced the number of taste buds in rodents [33] we could not confirm a relationship between the inflammatory markers IL-­6 (*r* = 0.05, *p* >0.05) and TNF­-α (*r* = −0.10, *p* >0.05) with the number or area of FP in our study. In addition, higher physical activity correlated with elevated IL-6 levels and sucrose recognition thresholds, yet neither variable correlated with FP number or area. These data suggest in summary, that (I) FP number and area are influenced in high exercise regimens by mechanisms that are independent of low-grade inflammation and (II) exercise-related inflammation affects sweet taste perception through mechanisms independent of papillae morphology. These mechanisms may include altered taste cell function, sweet receptor expression, the number of taste buds per FP, or signal transduction including neurotransmitter release by taste receptor cells and all should be identified in future studies. However, FP contain taste cells equipped with chemosensors for all taste qualities. Therefore, it cannot be excluded that the exercise-induced reduction in FP number and area may affect the perception of other taste modalities than sweet taste, such as salty or fatty.

The elevated IL-6 levels detected in highly active participants may result from multiple exercise-related mechanisms. Our salivary biomarker measurements cannot distinguish between localized oral inflammation versus systemic inflammation reflected in saliva; therefore, the elevated IL-6 could originate from local, systemic, or combined sources. Vigorous exercise could lead to temporary xerostomia (dry mouth conditions), potentially causing localized inflammation of salivary glands and contributing to elevated salivary IL-6 levels. This mechanism parallels primary Sjögren’s syndrome, where salivary gland inflammation and decreased salivary output are associated with impaired taste perception [71]. Additionally, exercise stress could directly activate inflammatory pathways within the oral cavity itself.

Previous research has shown that taste receptor cells express Toll-like receptors (TLR), which when activated by inflammatory stimuli, induce cytokine expression including IL-6 [72]. Exercise-induced stress in the oral cavity could also directly activate these TLRs, serving as a potential local source of the elevated salivary IL-6 levels observed in our study. This locally produced IL-6 and other inflammatory mediators could activate TLR signaling pathways, creating a self-amplifying inflammatory cascade that modulates taste cell signaling at the molecular level. This mechanism could operate separately from FP morphology, as FP number is independently influenced by body composition and leptin levels, explaining the lack of correlation between IL-6 and FP measures in our dataset.

## Summary and limitations

Our results show that physical activity is associated with different aspects of sweet taste perception. Compared to participants with a low activity level, participants who performed more than 6 h of intense physical activity per week showed decreases in sucrose recognition thresholds and FP, a higher consumption of sweet foods and increased cytokine levels, suggesting that physical activity may affect chemosensory sweet taste signaling via inflammatory processes. Multiple regression analyses controlling for age, sex, and BMI revealed that IL-6 independently contributes to the sucrose recognition threshold through inflammatory rather than metabolic pathways, while FP characteristics operate through independent mechanisms involving body composition and leptin.

These findings should be interpreted within the context and acknowledged limitations of our non-invasive study design. Salivary hormone levels may not always reflect plasma levels, potentially obscuring certain relationships. Although intensive physical activity was associated with reduced FP number and area, FP markers showed no relationship with sweet taste perception. However, the study design also limited our ability to investigate molecular chemosensory signaling changes which may provide further explanations.

While IL-6 significantly influences sweet taste sensitivity, this relationship explains only limited variance, suggesting additional factors are involved. Furthermore, our observational design cannot determine whether elevated IL-6 actively induces oral inflammation or serves as a marker of existing inflammatory processes. Future studies should examine chemosensory signaling at the molecular level using more invasive methods to determine taste receptor expression patterns. Additionally, studies should investigate whether specific exercise modalities (endurance vs. resistance training) differentially affect taste perception. In addition, our study focused exclusively on sweet taste perception, limiting conclusions about whether exercise-induced inflammatory responses specifically affect sweet taste or influence multiple taste modalities.

Despite these limitations, our results provide novel insights into the plasticity of sweet taste perception. We demonstrated that physically active individuals exhibit altered sweet taste perception and propose an underlying mechanism mediated by inflammatory processes. Given that taste is a critical determinant of dietary habits, these findings offer insight into the formation of nutritional behaviors in individuals engaging in high amounts of physical activity.

## Supplementary Information

Supplementary Material 1

## Data Availability

Mean values of the data are presented throughout the manuscript. Individual data are available from the corresponding author on reasonable request.

## Abbreviations

3-AFC: 3-Alternative Forced Choice
8-iso-PGF2α: 8-iso-prostaglandin F2α
BIA: Bio impedance analysis
BCM: body cell mass
CRP: C-reactive protein
FFQ: food frequency questionnaire
FP: fungiform papillae
GLP-1: glucagon-like peptide 1
IL-6: Interleukin 6
IPAQ: International Physical Activity Questionnaire
MET: Metabolic equivalent of task
PCA: Principal Component Analysis
PC1: Principal Component 1
PC2: Principal Component 2
RMR: Resting metabolic rate
TAS1R2/R3: Taste receptor type 1 member 2 / member 3
TLR: Toll-like receptor
TNF-α: tumor necrosis factor alpha
