# Supplementary material for: High physical activity is associated with decreased fungiform papillae area and number, elevated sucrose recognition thresholds, and increased IL-6 levels: an observational human study
Source: Nutr Metab (Lond). 2025 Nov 28;22:148. doi: 10.1186/s12986-025-01050-8 (PMC12670789; doi:10.1186/s12986-025-01050-8)
Supplement: Supplementary file 1 — Supplementary Material 1 [file 12986_2025_1050_MOESM1_ESM.docx]

**Supplementary Information**

**High physical activity is associated with decreased fungiform papillae area and number, elevated sucrose recognition thresholds, and increased IL-6 levels: An observational human study**

Isabella Kimmeswenger ^1,2^, Marlies Gaider ^2,3^, Kevin Doppelmayer ^1^, Jakob P. Ley ^4^ and Barbara Lieder ^1, 3, 5*^

^1^ Institute of Physiological Chemistry, Faculty of Chemistry, University of Vienna, 1090, Vienna, Austria

^2^ Vienna Doctoral School in Chemistry (DoSChem), University of Vienna, 1090, Vienna, Austria

^3^ Christian Doppler Laboratory for Taste Research, Faculty of Chemistry, University of Vienna, 1090, Vienna, Austria

^4^ Symrise AG, 37603, Holzminden, Germany

^5^ Institute of Clinical Nutrition, University of Hohenheim, 70599, Stuttgart, Germany

* Correspondence: Barbara Lieder, barbara.lieder@uni-hohenheim.de

***Supplementary Table S1.*** *Measured variables for total cohort and across physical activity groups. Values are depicted as mean ± SD. IL-6 = Interleukin 6, 8-iso-PGF2α ­ 8-iso-prostaglandin F2α, FP = fungiform papillae, BCM = body cell mass, FFQ = food frequency questionnaire, GLP-1 = glucagon-like peptide 1, TNF-α = tumor necrosis factor alpha*

|  | **Total cohort** | **≤ 2.5 h physical activity** | **≥ 6 h physical activity** |
| --- | --- | --- | --- |
| **Salivary IL-6 [pg/mL]** | 11.19 ± 4.71 | 9.77 ± 3.78 | 12.49 ± 5.14 |
| **Urinary 8-iso-PGF-α [pg/mg Creatinine]** | 896.7 ±1342 | 537.2 ± 479 | 1224 ± 1746 |
| **FP area [%]** | 11.90 ± 4.27 | 14.10 ± 3.68 | 9.89 ± 3.79 |
| **FP number** | 24.25 ± 6.6 | 28.06 ± 5.84 | 20.76 ± 5.22 |
| **Sucrose recognition threshold [g/L]** | 3.91 ± 1.82 | 3.25 ± 1.28 | 4.52 ± 2.03 |
| **BCM [%]** | 43.30 ± 5.05 | 42.13 ± 5.44 | 44.37 ± 4.48 |
| **Body fat [%]** | 20.77 ± 6.24 | 21.22 ± 6.66 | 20.36 ± 5.91 |
| **Sweet consumption [FFQ score]** | 7.21 ± 1.42 | 6.77 ± 1.27 | 7.60 ± 1.45 |
| **Sweet preference [factor score]** | 0 ± 1 | -0.17 ± 1.08 | 0.157 ± 0.91 |
| **Leptin [pg/mL]** | 10.82 ± 7.18 | 12.89 ± 8.08 | 8.88 ± 5.68 |
| **GLP-1 [pM]** | 0.55 ± 0.5 | 0.62 ± 0.53 | 0.48 ± 0.47 |
| **Glucagon [pg/mL]** | 2.95 ± 1.09 | 2.91 ± 0.79 | 2.99 ± 1.31 |
| **Serotonin [pg/sample]** | 3.29 ± 2.93 | 3.64 ± 3.45 | 2.97 ± 2.34 |
| **TNF-α [pg/mL]** | 21.34 ± 17.54 | 19.25 ± 14.44 | 23.26 ± 19.99 |

***Supplementary Table S2.*** *Multiple Linear Regression Analysis: Predictors for FP area. Model Statistics: Model 1: R² = 0.086, F(5,59) = 1.112, p = 0.364, n = 65. Model 2: R² = 0.085, F(5,58) = 1.076, p = 0.383, n = 64. BCM = body cell mass, BMI = body mass index*

| **Model** | **Predictor** | **β (unstandardized)** | **95 % CI** | **t-value** | **p-value** |
| --- | --- | --- | --- | --- | --- |
| **Model 1** | BCM [%] | -0.074 | [-0.601, 0.454] | -0.280 | 0.781 |
|  | Body fat [%] | 0.100 | [-0.344, 0.543] | 0.450 | 0.654 |
|  | Age [years] | 0.192 | [-0.051, 0.435] | 1.584 | 0.118 |
|  | Sex  [female = 1] | 0.769 | [-2.352, 3.890] | 0.493 | 0.624 |
|  | BMI | -0.327 | [-0.986, 0.332] | -0.993 | 0.325 |
| **Model 2** | Leptin [pg/mL] | -0.001 | [-0.156, 0.154] | -0.013 | 0.990 |
|  | Body fat [%] | 0.152 | [-0.086, 0.390] | 1.274 | 0.208 |
|  | Age [years] | 0.192 | [-0.059, 0.442] | 1.532 | 0.131 |
|  | Sex  [female = 1] | 0.684 | [-2.520, 3.888] | 0.427 | 0.671 |
|  | BMI | -0.366 | [-0.971, 0.239] | -1.211 | 0.231 |

***Supplementary Table S3.*** *Multiple Linear Regression Analysis: Predictors for FP number. Model Statistics: Model 1: R² = 0.254, F(5,59) = 4.011, p = 0.003, n = 65. Model 2: R² = 0.300, F(5,58) = 4.969, p < 0.001, n = 64. BCM = body cell mass, BMI = body mass index*

| **Model** | **Predictor** | **β (unstandardized)** | **95 % CI** | **t-value** | **p-value** |
| --- | --- | --- | --- | --- | --- |
| **Model 1** | BCM [%] | -0.056 | [-0.792, 0.680] | -0.151 | 0.880 |
|  | Body fat [%] | 0.621 | [0.002, 1.240] | 2.007 | 0.049 |
|  | Age [years] | 0.057 | [-0.282, 0.396] | 0.335 | 0.739 |
|  | Sex | 4.097 | [-1.260, 8.454] | 1.882 | 0.165 |
|  | BMI | -1.231 | [-2.150, -0.311] | -2.678 | 0.010 |
| **Model 2** | Leptin | 0.209 | [0.0003, 0.419] | 2.004 | 0.050 |
|  | Body fat [%] | 0.619 | [0.298, 0.941] | 3.860 | < 0.001 |
|  | Age [years] | 0.125 | [-0.213, 0.463] | 0.741 | 0.462 |
|  | Sex [female = 1] | 3.353 | [-0.970, 7.676] | 1.553 | 0.126 |
|  | BMI | -1.167 | [-1.984, -0.351] | -2.863 | 0.006 |

***Supplementary Table S4.*** *Multiple Ordinal Regression Analysis: Predictors for Sweet Taste Sensitivity (Sucrose Recognition Threshold). Model Statistics: AIC = 122.5, Residual Deviance = 110.5, Brant test: χ² = 1.60, p = 0.808, n = 65. IL-6 = Interleukin 6, BMI = body mass index*

| **Predictor** | **β (unstandardized)** | **95 % CI** | **t-value** | **p-value** |
| --- | --- | --- | --- | --- |
| **IL-6 [pg/mL** | 0.117 | [0.002, 0.236] | 1.929 | 0.045 |
| **Age [years]** | -0.053 | [-0.168, 0.062] | -0.901 | 0.368 |
| **Sex [male = 1]** | 0.019 | [-1.078, 1.115] | 0.034 | 0.973 |
| **BMI** | 0.038 | [-0.194, 0.270] | 0.322 | 0.748 |

***Supplementary Table S5.*** *Multiple Linear Regression Analysis: Predictors for sweet consumption. Model Statistics: R² = 0.082, F(4,60) = 1.342, p = 0.265, n = 65. BMI = body mass index*

| **Predictor** | **β (unstandardized)** | **95 % CI** | **t-value** | **p-value** |
| --- | --- | --- | --- | --- |
| **Sweet recognition threshold [g/L]** | 0.178 | [-0.021, 0.378] | 1.786 | 0.079 |
| **Age [years]** | 0.049 | [-0.032, 0.130] | 1.217 | 0.228 |
| **Sex [female = 1]** | -0.153 | [-0.938, 0.632] | -0.390 | 0.698 |
| **BMI [kg/m²]** | 0.079 | [-0.088, 0.245] | 0.946 | 0.348 |
